# Histone deacetylase regulates insulin signaling via two pathways in pancreatic β cells

**DOI:** 10.1371/journal.pone.0184435

**Published:** 2017-09-08

**Authors:** Yukina Kawada, Shun-ichiro Asahara, Yumiko Sugiura, Ayaka Sato, Ayuko Furubayashi, Mao Kawamura, Alberto Bartolome, Emi Terashi-Suzuki, Tomoko Takai, Ayumi Kanno, Maki Koyanagi-Kimura, Tomokazu Matsuda, Naoko Hashimoto, Yoshiaki Kido

**Affiliations:** 1 Division of Metabolism and Disease, Department of Biophysics, Kobe University Graduate School of Health Sciences, Kobe, Japan; 2 Division of Diabetes and Endocrinology, Department of Internal Medicine, Kobe University Graduate School of Medicine, Kobe, Japan; 3 Medical Technology Major, Faculty of Health Sciences Major, Kobe University Graduate School of Medicine, Kobe, Japan; 4 Department of Medicine, Columbia University Medical Center, New York, New York, United States of America; Broad Institute, UNITED STATES

## Abstract

Recent studies demonstrated that insulin signaling plays important roles in the regulation of pancreatic β cell mass, the reduction of which is known to be involved in the development of diabetes. However, the mechanism underlying the alteration of insulin signaling in pancreatic β cells remains unclear. The involvement of epigenetic control in the onset of diabetes has also been reported. Thus, we analyzed the epigenetic control of insulin receptor substrate 2 (IRS2) expression in the MIN6 mouse insulinoma cell line. We found concomitant IRS2 up-regulation and enhanced insulin signaling in MIN6 cells, which resulted in an increase in cell proliferation. The H3K9 acetylation status of the *Irs2* promoter was positively associated with IRS2 expression. Treatment of MIN6 cells with histone deacetylase inhibitors led to increased IRS2 expression, but this occurred in concert with low insulin signaling. We observed increased IRS2 lysine acetylation as a consequence of histone deacetylase inhibition, a modification that was coupled with a decrease in IRS2 tyrosine phosphorylation. These results suggest that insulin signaling in pancreatic β cells is regulated by histone deacetylases through two novel pathways affecting IRS2: the epigenetic control of IRS2 expression by H3K9 promoter acetylation, and the regulation of IRS2 activity through protein modification. The identification of the histone deacetylase isoform(s) involved in these mechanisms would be a valuable approach for the treatment of type 2 diabetes.

## Introduction

Type 2 diabetes mellitus is known to develop with increased peripheral insulin resistance or impaired insulin secretion from pancreatic β cells [1–3]. Recently, pancreatic β cell function was shown to be impaired early in the onset of diabetes, despite normal glucose tolerance [4, 5]. Furthermore, many reports have indicated that pancreatic β cell mass is also decreased in type 2 diabetic patients with impaired insulin secretion [6, 7].

This study focused on insulin signaling, an intracellular signaling pathway that regulates pancreatic β cell mass. Many studies have already reported that the insulin signaling pathway plays an important role in the regulation of pancreatic β cell mass [8–10]. Mice with a specific deletion of the insulin signaling-related *Pdk1* gene in pancreatic β cells showed a progressive decrease in pancreatic β cell mass that resulted in hypoinsulinemia and severe hyperglycemia [11]. In addition, the β cell-specific hyperactivation of mTORC1, a downstream effector of insulin signaling, results in enhanced β cell mass, hyperinsulinemia, and hypoglycemia at a young age [12]. These findings suggest that alterations of β cell insulin signaling have important consequences for β cell mass and insulinemia and can thus play an important role in the progression of type 2 diabetes. However, the molecular mechanisms underlying the pathophysiological alterations of insulin signaling in pancreatic β cells remain unclear.

Children with low birth weight reportedly have a high risk of developing type 2 diabetes later in life [13, 14]. Therefore, we previously generated and analyzed a low birth weight mouse model. In that study, a reduction of pancreatic β cell mass was observed at birth, followed by a rapid increase in pancreatic β cell mass. Using pancreatic β cell-specific *Pdk1* heterozygous knockout mice with low birth weight, insulin signaling activity in the islets was found to be involved in the compensatory postnatal expansion of β cell mass [15, 16]. In addition, starvation stress during fetal development is known to affect epigenetic control in several organs. The number of pancreatic β cells reportedly decreases later in life through the epigenetic control of the transcription factor *Pdx1* in pancreatic β cells [17]. However, there has been no report showing that insulin signaling was enhanced or attenuated through epigenetic control. These findings prompted us to consider the possibility that insulin signaling in pancreatic β cells may be regulated through epigenetic control.

This study was designed to examine the epigenetic control of pancreatic β cells. We analyzed the mechanism regulating insulin signaling during β cell proliferation using the MIN6 mouse insulinoma cell line and db/db mouse model of type 2 diabetes.

## Materials and methods

### Ethics statement

This study was approved by the animal ethics committee of Kobe University Graduate School of Medicine (Permit number P160102).

### Cell culture and transfection of siRNA

MIN6 cells were routinely maintained in Dulbecco’s modified Eagle’s medium (SIGMA) containing 15% heat-inactivated fetal calf serum, and were cultured at 37°C with 5% CO_2_. The medium was replaced every 3–4 days.

Insulin signaling and epigenetic control of insulin receptor substrate 2 (*Irs2*) transcription were analyzed in cells at a low passage frequency (passages 25–30), designated as low passage number (Lpn), and cells at a high passage frequency (passages 60–65), designated as high passage number (Hpn).

MIN6 cells were transfected with small interfering RNA (siRNA) for HDAC1 and control (SMARTpool; Dharmacon), as described previously [18].

### Incubation of MIN6 cells with enzyme inhibitors

For the analysis of epigenetic control and post-translational modifications, MIN6 cells were exposed to 50 μM LY294002 (Wako) for 8 h, 10 ng/mL apicidin (Enzo Life Sciences) for 24 h, 10 μM tubacin (Sigma-Aldrich) for 24 h, 3 μM 5-aza-2′-deoxycytidine (5-aza-dC) (Sigma-Aldrich) for 72 h, 100 nM trichostatin A (TSA) (Sigma-Aldrich) for 24 h, and 5 μM suberoylanilide hydroxamic acid (SAHA) (Cayman Chemical) for 24 h on the day following seeding.

### Proliferation assay

The proliferation rates (doubling times) of the Lpn and Hpn MIN6 cells were measured by monitoring their growth over consecutive 24 h time periods on days 2–4. The cells were seeded at 10^5^ cells/well and incubated at 37°C/5% CO_2_. Three wells were trypsinized and counted on each of the 3 days. The cell number was the average number of 4 or more sections counted using a Burker-Turk counting chamber for each well. Cell doubling times were calculated by plotting cell number against time.

### Cell cycle analysis

For cell cycle analysis, a CycleTEST PLUS DNA Reagent Kit (Becton Dickinson) was used. After the indicated treatment, the cells were washed with ice-cold phosphate-buffered saline and collected. According to the manufacturer’s protocol to isolate and stain cell nuclei, the samples were then filtered using a 35-μm cell strainer, and DNA content was stained and determined by flow cytometry. All analyses were carried out on a FACSCalibur using CellQuest Software (Becton Dickinson).

### Mice

Lepr^+/-^ (db/m) and Lepr^-/-^ (db/db) mice on a C57BL/KsJ background were obtained from CLEA, Inc. The animals were maintained in a 12 h light/12 h dark cycle and fed normal chow, as described previously [19].

### Immunoblot analysis

Lysates of isolated islets and MIN6 cells were prepared as described previously [20] and probed with antibodies to PCNA (Dako), β-actin (Sigma-Aldrich), phospho-Akt (Thr308), phospho-Akt (Ser473), Akt, phospho-p70 S6 kinase, p70 S6 kinase, phospho-S6, S6, phospho-GSK3β, GSK3β, IRS2, phospho-CREB, and CREB (Cell Signaling).

### Real-time RT-PCR analysis

Total RNA was extracted from MIN6 cells using an RNeasy Kit (QIAGEN). RNA was subjected to reverse transcription (RT), and real-time polymerase chain reaction (PCR) analysis was performed as described previously [18]. The cDNA synthesized from the RNA was analyzed using a sequence detector (model 7500; Applied Biosystems) with specific primers and SYBR Green PCR Master Mix (QIAGEN). The relative abundance of each mRNA was normalized to the mRNA of the housekeeping gene cyclophilin A. Primers (sense and antisense, respectively) were as follows: cyclophilin, 5′-CAGACGCCACTGTCGCTTT-3′ and 5′-TGTCTTTGGAACTTTGTCTGCAA-3′; *Ir*, 5′-TTTGTCATGGATGGAGGCTA-3′ and 5′-CCTCATCTTGGGGTTGAACT-3′; *Irs1*, 5′-CCCACAGCAGATCATTAACC-3′ and 5′-AGAGACGAAGATGCTGGTGC-3′; *Irs2*, 5′-AGTCCCACATCGGGCTTGAAG-3′ and 5′-GGTCTGCACGGATGACCTTAG-3′; and *PI3Kp85a*, 5′-CCTTGTCCGGGAGAGCAGTA-3′ and 5′-TTGACTTCGCCGTCTACCACT-3′.

### DNA bisulfite modification, combined bisulfite restriction analysis (COBRA), and direct sequencing

Genomic DNA was extracted from MIN6 cells using a DNeasy Blood and Tissue Kit (QIAGEN) following the manufacturer’s instructions. The quality and quantity of genomic DNA was assessed using a NanoDrop ND1000 spectrophotometer (Thermo Scientific).

Bisulfite treatment of DNA was performed using an EpiTect Bisulfite Kit (QIAGEN) according to the manufacturer’s instructions. For methylation-specific PCR, bisulfite-modified DNA (2 μL) was amplified in a volume of 20 μL using specific primers targeting the *Irs2* gene (including the transcription factor binding sites) and 1 U of Ex Taq DNA Polymerase Hot-Start Version (TaKaRa), according to the manufacturer’s instructions. The primer sequences were 5′-GGGAATTTGATAAGTGAATGG-3′ and 5′-TCCCACTAACTAACCCCAAA-3′. The following amplification conditions were used: 98°C for 1 min, followed by 40 cycles of 98°C for 10 s, 55°C for 30 s, and 72°C for 1 min, then 72°C for 7 min. The amplification products were digested with *Bst*UI (CGCG) (Biolabs) at 37°C, and visualized on 2.0% agarose gels with ethidium bromide staining.

For direct sequencing, bisulfite-modified DNA was amplified using the primers for COBRA analysis and separated on 2.0% agarose gels. PCR products were purified with a QIAquick Gel Extraction Kit (QIAGEN) and cloned by using a TOPO TA Cloning Kit (Thermo Scientific). The clones were sequenced on an ABI 310 Sequencer with a BigDye^®^ Terminator Cycle Sequencing Kit (Applied Biosystems). Sequenced products were analyzed with Sequence Scanner Software v5.3.1 (Applied Biosystems).

### Chromatin immunoprecipitation (ChIP) analysis

ChIP analysis was performed using a Magna ChIP G Kit (Millipore). In brief, isolated islets and MIN6 cells were fixed with 1% formaldehyde for 30 min at room temperature and then subjected to ultrasonic disruption in a solution containing a protease inhibitor cocktail. The lysates were centrifuged to remove debris, diluted 1:10 with a solution containing 1% Triton X-100, 2 mM EDTA, 20 mM Tris-HCl (pH 8.1), and 150 mM NaCl, and incubated for 2 h at 4°C with protein G–Sepharose beads. The beads were removed by centrifugation, and the supernatants were subjected to immunoprecipitation by incubation at 4°C overnight with antibodies to acetylated H3K9/K14 (Cell Signaling) or with normal mouse immunoglobulin G (Santa Cruz Biotechnology) followed by 1 h incubation with protein G–Sepharose. The precipitates were washed and then subjected to extraction for 4 h at 65°C with 1% SDS in 100 mM NaHCO_3_. Proteins were digested with proteinase K, and the remaining DNA was purified using a QIAquick PCR Purification Kit (QIAGEN) and subjected to PCR with *Irs2*-specific primers (sense, 5′-CTATTACATCCAGAACAGGCG-3′; antisense, 5′-ATGGCAGCTCGGTGCCTTTT-3′).

### Immunoprecipitation analysis

IRS2 protein modifications were examined with an immunoprecipitation assay. Briefly, to block nonspecific background, protein G agarose beads (GE Healthcare), at a final concentration of 5% (v/v), were incubated with 500 μg total lysate from MIN6 cells in lysate buffer at 4°C for 2 h. The resulting mixture was centrifuged at 5000 rpm for 3 min, and the harvested supernatant was incubated with 4 μg IRS2 antibody with gentle agitation at 4°C overnight. Protein G beads were then added to a final concentration of 2.5% (v/v). The mixture was further incubated for 1 h at 4°C with gentle agitation and centrifuged at 5000 rpm for 3 min. The supernatant was mixed with SDS sample buffer and subjected to SDS-PAGE. The harvested beads were washed 3 times with TBS-T, re-suspended in SDS sample buffer, heat denatured, and subjected to SDS-PAGE. The proteins resolved by SDS-PAGE were profiled by immunoblot analysis using antibodies against IRS2, acetylated-lysine, phospho-tyrosine (Cell Signaling), and β-actin (Sigma-Aldrich) for definitive identification and quantification.

### Insulin secretion assay

Insulin secretion from MIN6 cells was measured after a 30-min incubation in Krebs-Ringer-bicarbonate-4-(2-hydroxyethyl)-1-piperazine ethanesulfonic acid (HEPES) buffer (140 mM NaCl, 3.6 mM KCl, 0.5 mM NaH_2_PO_4_, 0.5 mM MgSO_4_, 1.5 mM CaCl_2_, 2 mM NaHCO_3_, 10 mM HEPES, and 0.1% bovine serum albumin; pH 7.4) containing the indicated stimulators. Insulin content was determined after extraction with acid ethanol.

### Statistical analysis

Data are presented as mean ± standard error of the mean (SEM). The significance of differences between independent means was assessed using the Mann-Whitney U-test. A *P*-value < 0.05 was considered statistically significant.

## Results

### Insulin-stimulated signal transduction and IRS2 expression are increased in MIN6 cells at high passage numbers

**Fig 1:**
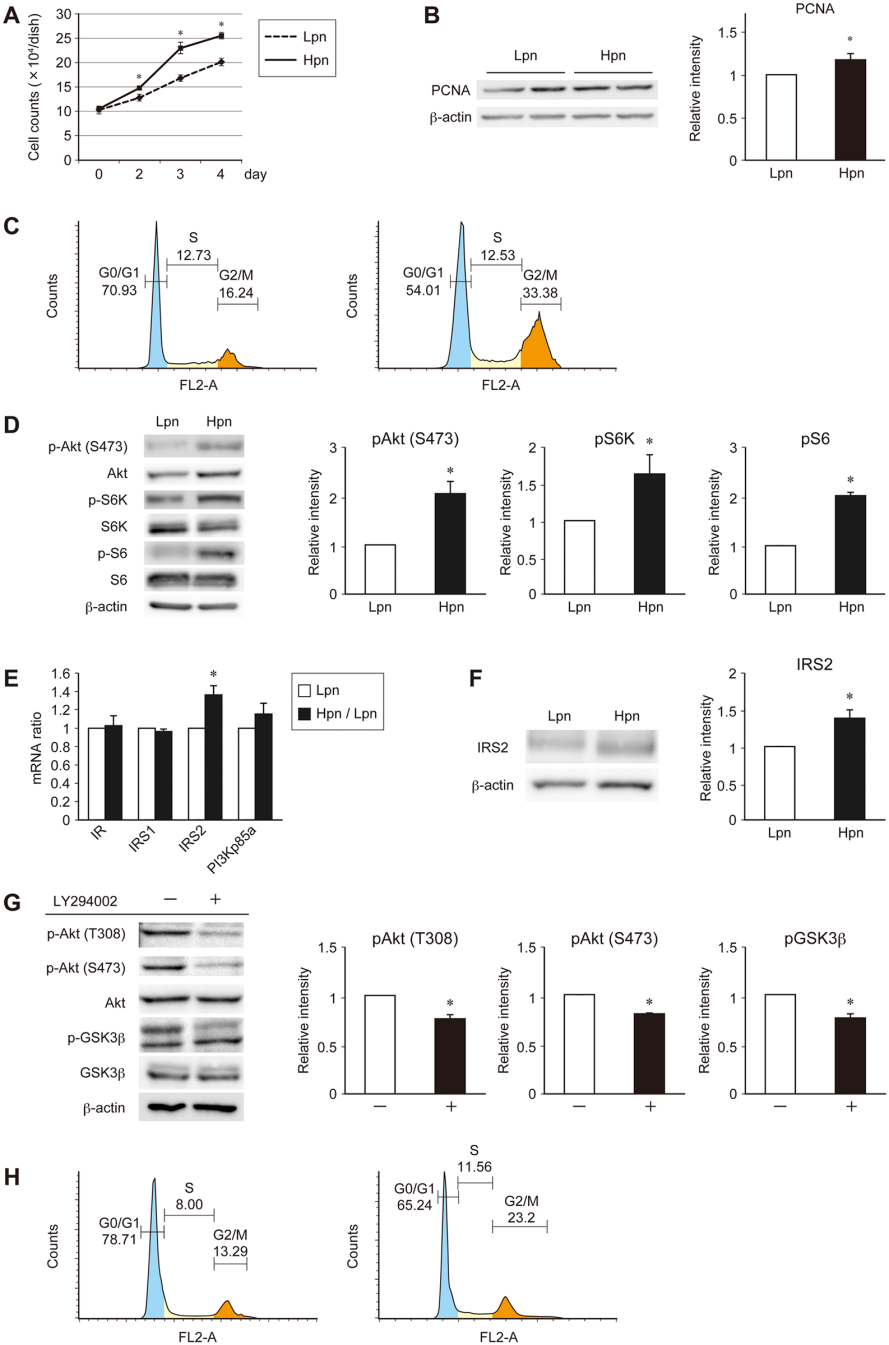
Analysis of the proliferation potency of MIN6 cells at different passage frequencies. (A) Proliferation data comparing Lpn (dotted line) and Hpn (straight line) MIN6 cells. (B, D) Immunoblot analysis of PCNA (B) and insulin signaling proteins (D) in Lpn and Hpn MIN6 cells. Representative (left) and quantitative (right) data are shown. (C) Representative cell cycle analysis in Lpn (left) and Hpn (right) MIN6 cells. (E) Quantitative real-time PCR analysis of insulin signaling molecule mRNA expression in Lpn (white bars) and Hpn (black bars) MIN6 cells. (F) Immunoblot analysis of IRS2 in Lpn and Hpn MIN6 cells. Representative (left) and quantitative (right) data are shown. (G, H) Representative (left) and quantitative (right) immunoblot analysis of insulin signaling proteins (G) and representative cell cycle analysis (H) in Hpn cells with or without LY294002. Data are represented as the mean ± SEM for 4 (A), and 5 (B–G) independent experiments. *P < 0.05.

To investigate the epigenetic regulation of insulin signaling, we analyzed the MIN6 mouse pancreatic β cell line with growth rate variation, considering that insulin signaling plays an important role in pancreatic β cell proliferation. The proliferation rate of MIN6 cells reportedly increases with passage number [21]. To exclude the indirect effects of cytotoxicity using gene knockdown or drug reagents, the experiment was performed using 2 groups of MIN6 cells with different passage frequencies (Lpn: passages 25–30; Hpn: passages 60–65). As expected, the growth rate of the Hpn group was increased (Fig 1A), along with the increased expression of the PCNA proliferation marker (Fig 1B). In cell cycle analysis, as shown previously [22], the G2 / M phase ratio was increased and the G0 / G1 phase ratio was decreased in the Hpn group (Fig 1C). Insulin signaling was also increased in the Hpn group (Fig 1D). Furthermore, the expression levels of the upstream molecules involved in insulin signaling were analyzed to unravel the mechanism implicated in the observed increase in insulin signaling. The expression of IRS2 in the Hpn group was increased at both the mRNA (Fig 1E) and protein level (Fig 1F). In order to confirm that this increase of insulin signaling due to increased IRS2 expression was responsible for the proliferation of MIN6 cells, we conducted an experiment using LY294002, an inhibitor of PI3K, which is located downstream of IRS2, in the Hpn group. As a result, the insulin signaling that was enhanced in the Hpn cell group was attenuated, and the proportion of cells in the G0 / G1 phase was also increased in cell cycle analysis (Fig 1G and 1H). These data suggest that insulin-stimulated signal transduction in high passage MIN6 cells plays an important role in cell proliferation, which was regulated through increased IRS2 expression.

### IRS2 expression in pancreatic β cells is increased as a result of H3K9 acetylation of the *Irs2* promoter region

Transcription is controlled by changes in nucleotide sequence, transcriptional regulatory factors, and epigenetic regulation, which affects gene expression without a change in the nucleotide sequence. DNA methylation of gene promoters is known to repress transcription, while it can also lead to increased expression if the binding sites of repressive factors are specifically methylated, while other mechanisms, such as histone modifications, regulate transcription through the control of chromatin structure.

**Fig 2:**
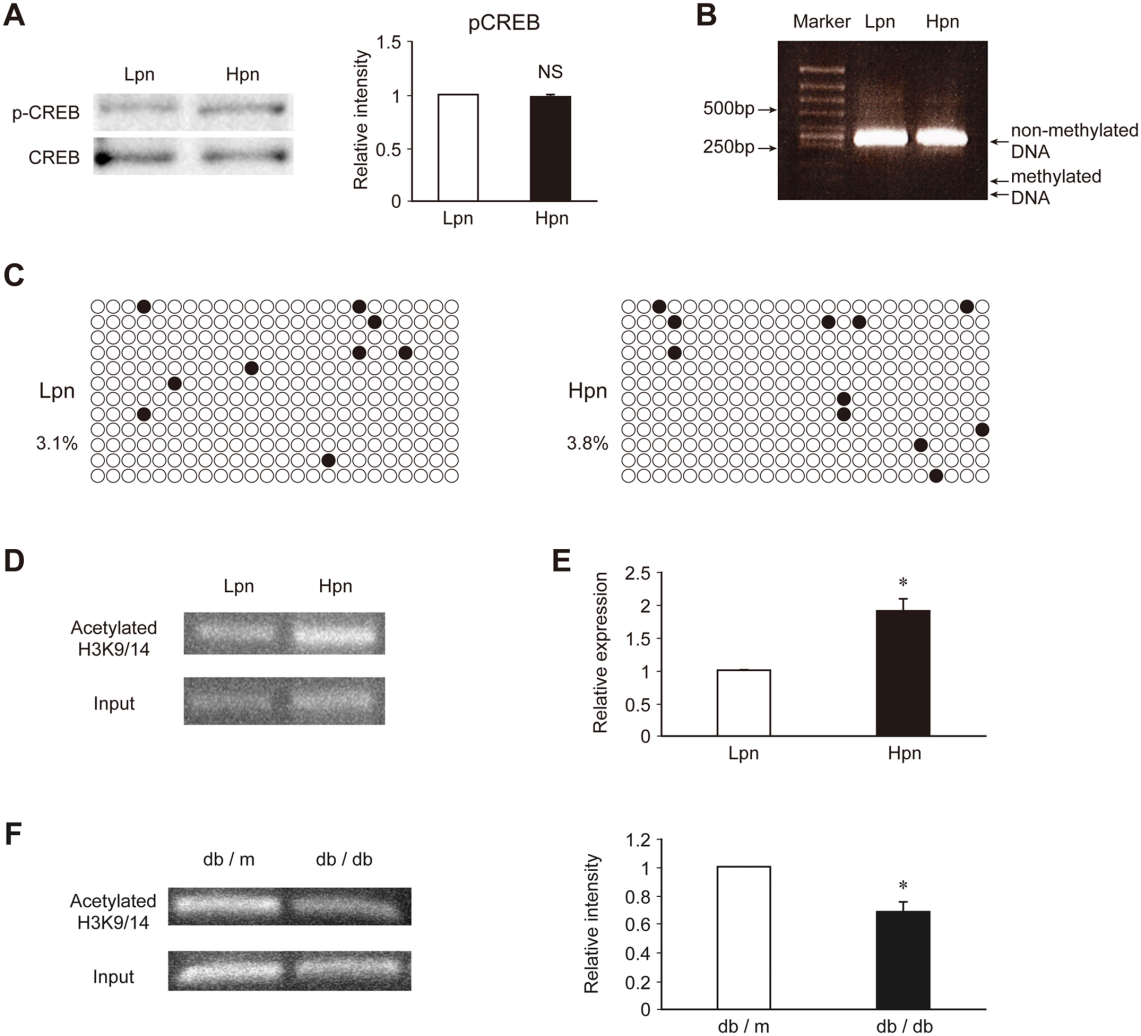
Analysis of the epigenetic regulation of insulin signaling molecules in pancreatic β cells. (A) Immunoblot analysis of the transcription factor CREB in Lpn and Hpn MIN6 cells. Representative (left) and quantitative (right) data are shown. (B) Combined bisulfite restriction analysis of DNA methylation. (C) Bisulfite sequence of the *Irs2* promoter region in Lpn and Hpn MIN6 cells. Black circles indicate methylated CpG sites and white circles indicate non-methylated CpG sites. Percentage is the ratio of methylated CpG sites. (D) Representative ChIP analysis of H3K9/14 histone acetylation of the *Irs2* promoter region in Lpn and Hpn MIN6 cells. (E) ChIP-qPCR of H3K9/14 histone acetylation of the *Irs2* promoter region in Lpn and Hpn MIN6 cells. (F) ChIP analysis of H3K9/14 histone acetylation of the *Irs2* promoter region and immunoblot analysis of IRS2 in islets isolated from db/db or control db/m mice at 10 weeks of age. Representative (left) and quantitative (right) data are shown. Data are represented as the mean ± SEM for 5 (A–F) independent experiments. *P < 0.05.

To explore the mechanism involved in the increase in IRS2 expression observed in the Hpn group, the transcriptional regulation mechanisms described above were analyzed. First, protein expression and phosphorylation of CREB, a known transcriptional regulator of the *Irs2* gene [23], were analyzed. However, no difference was observed in the expression or activity of CREB between the Lpn and Hpn groups (Fig 2A).

Next, DNA methylation, which is an epigenetic modification known to control gene expression, was examined using the bisulfite COBRA method and bisulfite sequencing. DNA methylation in the *Irs2* promoter region was observed at very low levels in both the Lpn and Hpn groups by COBRA (Fig 2B). In addition, bisulfite sequence also revealed hypomethylation of the *Irs2* promoter in both groups (Fig 2C).

ChIP analysis of H3K9 acetylation in the *Irs2* promoter region was also performed (S1 Fig). A significant enhancement of H3K9 acetylation was observed in the Hpn group (Fig 2D and 2E). H3K9 acetylation is an epigenetic mark associated with transcriptional enhancement; thus, these results indicate that histone modification was involved in the elevated IRS2 expression observed in the Hpn group. To investigate whether this mechanism contributed to the change in insulin signaling in pancreatic β cells under diabetic conditions *in vivo*, we conducted similar studies on isolated islets from db/db mice, a diabetic mouse model. Indeed, IRS2 expression in islets from db/db mice was reported previously to be decreased [24]. Therefore, ChIP analysis was performed, and a decrease in H3K9 acetylation was observed in the *Irs2* promoter region (Fig 2F). However, in the 10-week-old db/db mice we used for these experiments, the proportion of α cells is greater than that of β cells in the islets, and it has also been reported that IRS2-Akt signaling plays an important role in the proliferation of α cells [25]. Since the possibility that these data could indicate the mechanism underlying the control of IRS2 expression in α cells of db/db mice cannot be excluded, further investigation is necessary.

### IRS2 expression is increased in MIN6 cells treated with the histone deacetylase (HDAC) inhibitors TSA or SAHA

**Fig 3:**
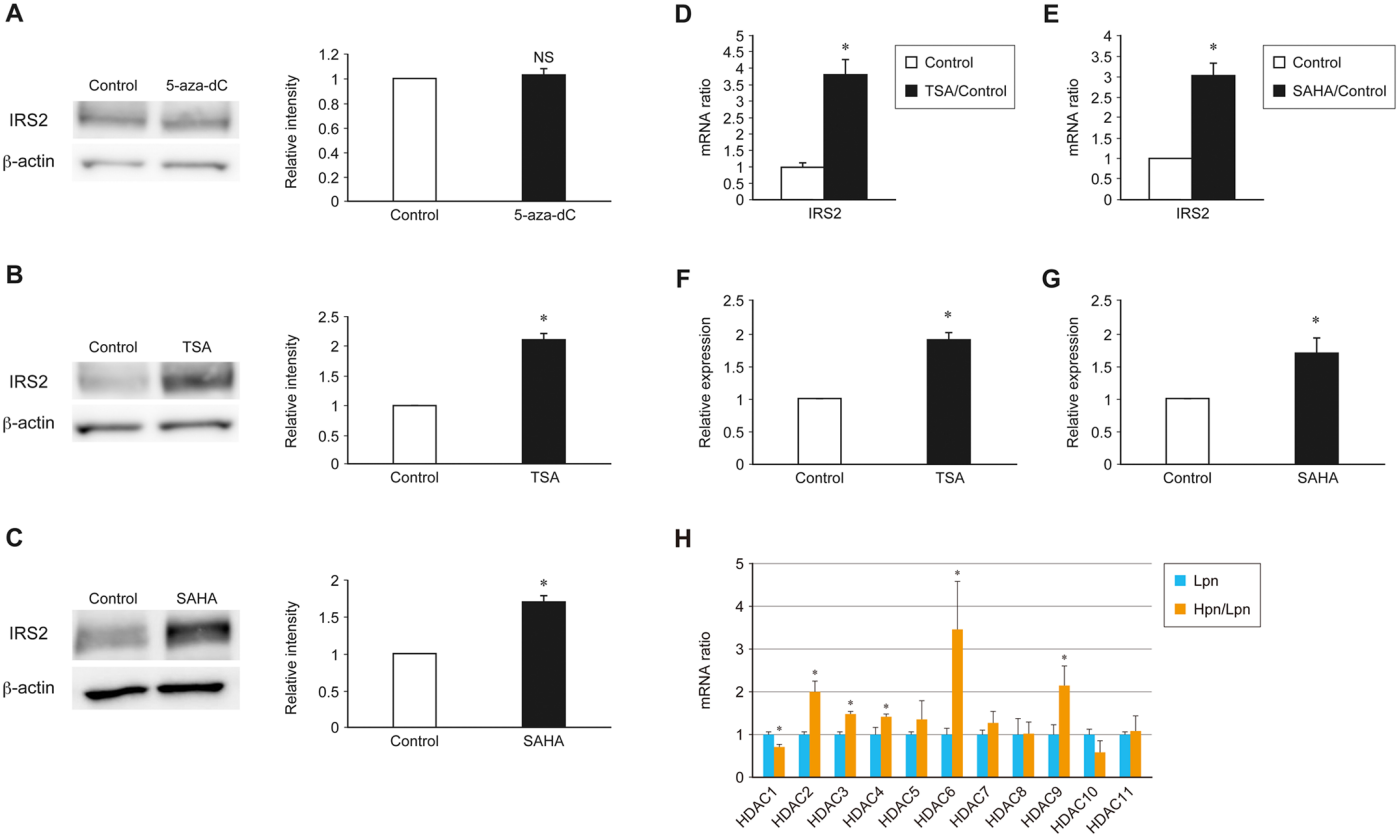
Effects of specific HDAC inhibitors on the expression of IRS2 in MIN6 cells. (A–C) Immunoblot analysis of IRS2 in MIN6 cells treated with the DNA methylation inhibitor 5-aza-dC (A) or the class I and class II HDAC inhibitors TSA (B) and SAHA (C). Representative (left) and quantitative (right) data are shown. (D, E) Quantitative real-time PCR analysis of *Irs2* mRNA expression in control MIN6 cells (white bars) and those treated with TSA (D) or SAHA (E) (black bars). (F, G) ChIP-qPCR of H3K9/14 histone acetylation of the *Irs2* promoter region in MIN6 cells with TSA (F) or SAHA (G). (H) Quantitative real-time PCR analysis of HDAC isoforms in Lpn and Hpn MIN6 cells. Data are represented as the mean ± SEM for 5 (A–H) independent experiments. *P < 0.05.

To confirm further the role of epigenetic control mechanisms in IRS2 expression and insulin signaling activity, we examined whether DNA methylation or HDAC inhibitors altered IRS2 expression in MIN6 cells. No change was observed in the expression of IRS2 in MIN6 cells treated with the DNA methylation inhibitor 5-aza-dC (Fig 3A). This result was consistent with the observed low level of DNA methylation in the *Irs2* promoter region (Fig 2B and 2C). Conversely, in MIN6 cells treated with TSA or SAHA, which are both class I and class II HDAC inhibitors, a significant up-regulation of IRS2 expression was observed both at the protein and mRNA level (Fig 3B–3E). Analysis by ChIP-quantitative PCR (qPCR) revealed that both TSA and SAHA enhanced H3K9 acetylation in the IRS2 promoter (Fig 3F and 3G). HDACs are responsible for the deacetylation of histones or specific molecules. These data indicate that the transcription of *Irs2* was enhanced as a result of an increase in histone acetylation induced by HDAC inhibition by TSA or SAHA.

Furthermore, in order to identify the HDAC isoform responsible for the differences in IRS2 expression level and insulin signaling between the Lpn and Hpn groups, we examined the HDAC isoforms in each group of cells by real-time PCR. As a result, only the expression of HDAC1 was significantly reduced in the Hpn group (Fig 3H). Since HDAC1 is localized mainly in the nucleus and it was considered that this may contribute to H3K9 acetylation in the IRS2 promoter region, the Lpn group was treated with apicidin, which is an HDAC class I-specific inhibitor including HDAC1. As a result, IRS2 expression was significantly increased, similarly to the Hpn group (S2A and S2B Fig). In addition, ChIP-qPCR analysis confirmed that H3K9 acetylation of the IRS2 promoter region was enhanced (S2C Fig). For further specific experiments, similar studies were conducted using MIN6 cells transfected with HDAC1 siRNA. As a result, the expression of IRS2 was elevated and the insulin signal was increased in HDAC1 knockdown cells (S2D and S2E Fig). In addition, cell cycle analysis confirmed that the proportion of cells in the G2 / M phase was increased (S2F Fig).

Conversely, when MIN6 cells were treated with tubacin, which is a specific inhibitor of HDAC6, there was no change in H3K9 acetylation in the IRS2 promoter region or IRS2 expression (S2G and S2H Fig). These results suggest that the reduction of HDAC1 expression may contribute to H3K9 acetylation in the IRS2 promoter region of Hpn MIN6 cells. In addition, an insulin secretion assay was performed using Lpn MIN6 cells treated with apicidin or HDAC1 siRNA. As a result, a decrease in insulin secretion by high glucose load was shown with apicidin; conversely, in HDAC1 knockdown cells, an increase in insulin secretion was observed (S2I and S2J Fig).

### Phosphorylation of IRS2 is down-regulated through lysine acetylation in MIN6 cells, leading to a decrease in insulin signaling

**Fig 4:**
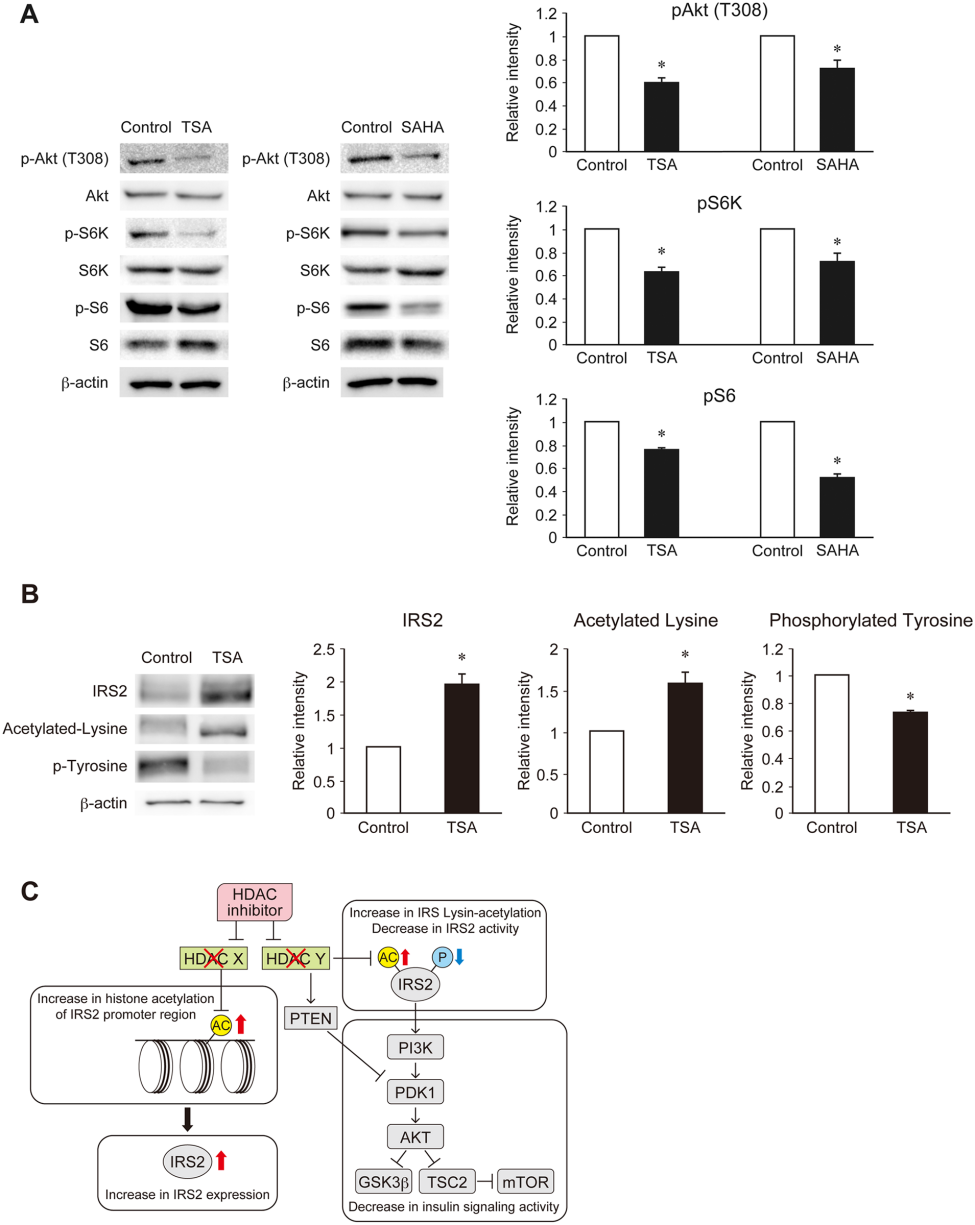
Effects of specific HDAC inhibitors on insulin signaling through IRS2 lysine acetylation in MIN6 cells. (A) Immunoblot analysis of insulin signaling proteins in MIN6 cells treated with TSA or SAHA. Representative (left) and quantitative (right) data are shown. (B) Immunoblot analysis of IRS2, acetylated lysine, and phosphorylated tyrosine in MIN6 cells treated with TSA after protein interaction analysis using antibodies to IRS2. Representative (left) and quantitative (right) data are shown. (C) Proposed model of HDAC regulation of IRS2 expression and activity in β cells. Data are represented as the mean ± SEM for 5 (A, B) independent experiments. *P < 0.05.

We next examined whether insulin signaling was increased following the up-regulation of IRS2 by the HDAC inhibitors TSA or SAHA in MIN6 cells. However, insulin signaling activity was significantly decreased in MIN6 cells treated with TSA or SAHA (Fig 4A). One possible reason for this is that lysine acetylation is a post-translational modification of IRS2 protein. Indeed, lysine acetylation of IRS2 protein was reported to reduce tyrosine phosphorylation in neuronal cells [26]. Therefore, IRS2 post-translational modifications were examined in MIN6 cells using immunoprecipitation analysis. An increase in lysine acetylation and a decrease in tyrosine phosphorylation of IRS2 protein were observed (Fig 4B). However, a recent report indicated that TSA treatment promotes PTEN acetylation, leading to a decrease in Akt signaling and downstream molecules [27]. Thus, the reduction of insulin signaling induced by TSA/SAHA may also be dependent on PTEN acetylation.

From the results of this study, a model was constructed to describe the mechanism controlling insulin signaling in pancreatic β cells (Fig 4C). In this model, if one isoform (X) of an HDAC is inhibited, the histone acetylation of the IRS2 promoter region and IRS2 transcriptional activity will be enhanced; thus, the expression of IRS2 will be increased. On the basis of our findings, we consider that isoform X is HDAC1. However, if another isoform (Y) of an HDAC is inhibited, the lysine acetylation of IRS2 will be enhanced, resulting in the suppression of IRS2 tyrosine phosphorylation. In addition, PTEN will be activated via lysine acetylation following HDAC inhibition, leading to a reduction in downstream insulin signaling activity.

## Discussion

In this study, the mechanisms controlling IRS2-dependent insulin signaling were analyzed using the db/db mouse model of type 2 diabetes and the MIN6 mouse insulinoma cell line. Our data suggest that insulin signaling in pancreatic β cells is controlled by two mechanisms involving HDACs: one mechanism acting on *Irs2* expression at the transcriptional level through epigenetic control, and the other affecting IRS2 activity at the post-transcriptional level through protein acetylation.

A decrease in insulin signaling reportedly results in the inhibition of gluconeogenesis suppression and hepatic steatosis in the liver and a reduction of glucose uptake in muscle, suggesting that insulin signaling is important in glucose homeostasis in the body [28–30]. Moreover, our study indicates that insulin signaling also plays an important role in the regulation of pancreatic β cell proliferation. However, the condition in which insulin signaling is increased or decreased in pancreatic β cells remains unclear. In this study, the mechanisms controlling insulin signaling in pancreatic β cells were revealed.

Continuous subculturing of MIN6 cells results in an increase in their proliferation rate; however, the mechanism of this increase remains unclear [21]. In contrast, several groups have reported potential mechanisms for the marked decrease in glucose-stimulated insulin secretion in high-passage MIN6 cells [31, 32]. Yamato et al. found that changes in DNA methylation are able to regulate the expression of specific genes in high-passage MIN6 cells that show reduced glucose-stimulated insulin secretion [31]. In this study, we confirmed a decrease in insulin secretion stimulated with high glucose by treating MIN6 cells with an HDAC class I-specific inhibitor; however, it was confirmed that insulin secretion was enhanced by HDAC1 siRNA (S2F Fig). This difference may be due to inhibitor toxicity or the effect of inhibiting the two remaining class I HDACs, HDAC2 and HDAC3. Insulin secretion is reportedly enhanced by inhibition of HDAC1, although this was observed under a condition of cytokine load [33]. Since HDAC1 inhibition causes an increase in IRS2 expression, it is considered that cell proliferation and enhancement of insulin secretion may also be mediated through IRS2. Since the expression of HDAC1 is decreased in Hpn cells, these findings suggest that MIN6 cells accumulate epigenetic changes as the passage number grows, leading to an increase in their proliferation rate, although this mechanism cannot explain the decline in insulin secretion.

Recently, the involvement of epigenetic control in the onset of so-called lifestyle-related diseases, such as diabetes, hyperlipidemia, high blood pressure, obesity, and metabolic syndrome, has been the focus of many studies. In the field of diabetes, the expression of PPAR-α, PEPCK, and GR in the liver after maturation is reportedly regulated through DNA methylation in low birth weight mice born with intrauterine growth delay [34]. More importantly, the expression of *Pdx1* in pancreatic β cells was shown to be regulated through changes in H3 acetylation and DNA methylation induced by intrauterine growth delay [17]. Furthermore, we recently reported that a mutation in the *Kcnq1* gene, a susceptibility gene for type 2 diabetes, reduced pancreatic β cell mass by epigenetic modulation [35].

HDACs catalyze the deacetylation of acetylated lysine residues of histone terminal regions. Human HDACs are classified into 4 groups: class I (HDAC1, 2, 3, and 8), class II (HDAC4, 5, 6, 7, 9, and 10), class III (SIRT1–7, known as sirtuins), and class IV (HDAC11). The localization of these 18 HDACs is regulated through nuclear export signals and nuclear localization signals in the gene sequences, suggesting that class I HDACs, except for HDAC3, are localized in the nucleus and the others can move between the nucleus and cytoplasm [36].

In this study, it was considered that HDAC X, acting on histone acetylation of the *Irs2* promoter region, is localized in the nucleus, and that HDAC Y, acting on lysine acetylation as a protein modification of IRS2, is localized in the cytoplasm. In addition, the expression level of each HDAC isoform in the brain and activity level in the kidney were reportedly altered in the diabetic condition [37–40]. HDAC4, 5, and 9 were reported to be involved in the differentiation of pancreatic β cells [41]. Several investigations using human pancreatic islets have also been reported, and HDAC1 expression is enhanced in pancreatic islets isolated from type 1 diabetic children, while the expression of HDAC2 and HDAC3 was decreased [33]. It has also been reported recently that HDAC7 expression is increased in the pancreatic islets of type 2 diabetic patients and the expression of Tcf7l2 is promoted independently of histone acetylation to suppress insulin secretion [42]. Here, it was revealed that there is a difference in the expression level of an HDAC isoform in MIN6 cells with an increase of passage number. In the Hpn group, only the expression of HDAC1 was decreased, and HDAC1 is known to localize to the nucleus. Therefore, HDAC X, which controls H3K9 acetylation in the IRS2 promoter region, is considered to be HDAC1. Conversely, HDAC Y, which deacetylates the lysine residues of IRS2, remains to be identified. Lysine acetylation of p53 and SREBP1a inhibits ubiquitination [43, 44], and lysine acetylation of FOXO and PGC1α promotes their auto-phosphorylation [45, 46]. With regard to the post-translational modifications of IRS2, lysine acetylation of IRS2 has been shown to reduce tyrosine phosphorylation in neuronal cells [26], but there has been no report regarding the post-translational modifications of IRS2 in pancreatic β cells. Therefore, further studies are required to define the mechanisms involved.

In this study, in order to confirm the control of IRS2 expression using only pure pancreatic β cells and to obtain reliable data from the ChIP assay, we needed a large amount of chromatin, so we focused our experiments on MIN6 cells. In future, in order to elucidate the role of HDACs *in vivo*, it is necessary to confirm the reproducibility of our findings in pancreatic islets. Currently, HDAC inhibitors are used in the clinical setting as anti-cancer (vorinostat, an inhibitor of HDAC1, 2, 3, and 6) or psychotropic (VPA, an inhibitor of HDAC1, 2, 3, and 8) drugs. Interestingly, cancer patients treated with HDAC inhibitors develop hyperglycemia as a side effect [47, 48], suggesting that HDAC inhibition may reduce insulin signaling in some organs. Our results provide a mechanism by which HDAC inhibition may impair glucose tolerance. These findings suggest that it is necessary to select more specific HDAC inhibitors to provide a protective effect for pancreatic β cells.

In summary, our study indicates that insulin signaling in pancreatic β cells is regulated by HDACs via two pathways: the epigenetic control of *Irs2* expression and the regulation of IRS2 activity through protein modification. Since IRS2 reportedly plays a very important role in pancreatic β cell proliferation [49], the identification of the HDAC isoforms involved in these mechanisms may be a valuable approach for the treatment of type 2 diabetes.

## Supporting information

S1 Fig IRS2 promoter region of mice. Arrows show the primers for ChIP analysis of H3K9/14 acetylation.
(TIF)

S2 Fig Effects of the specific HDAC inhibitors apicidin and tubacin and HDAC1 siRNA on the expression of IRS2 in MIN6 cells. (A) Immunoblot analysis of IRS2 in MIN6 cells treated with the HDAC class I-specific inhibitor apicidin. (B) Quantitative real-time PCR analysis of *Irs2* mRNA expression in control MIN6 cells (white bars) and those treated with apicidin (black bars). (C) ChIP-qPCR of H3K9/14 histone acetylation of the *Irs2* promoter region in MIN6 cells treated with apicidin. (D, E) Immunoblot analysis of IRS2 (D) and insulin signaling proteins (E) in MIN6 cells treated with HDAC1 siRNA. (F) Representative cell cycle analysis in Lpn MIN6 cells with (right) or without (left) HDAC1 siRNA. (G, H) ChIP-qPCR of H3K9/14 histone acetylation of the *Irs2* promoter region (G) and quantitative real-time PCR analysis of *Irs2* mRNA expression (H) in MIN6 cells treated with the HDAC6-specific inhibitor tubacin. (I) Insulin secretion in response to the indicated concentrations of glucose from Lpn MIN6 cells with or without apicidin. (J) Insulin secretion in response to the indicated concentrations of glucose from Lpn MIN6 cells with or without HDAC1 siRNA. Data are represented as the mean ± SEM for 5 (A–H) and 6 (I, J) independent experiments. *P < 0.05.
(TIF)

S3 Fig Immunoblot analysis of original blots. (TIF)
