# Supplementary figures and images for: Histone deacetylase regulates insulin signaling via two pathways in pancreatic β cells
Source: PLoS One. 2017 Sep 8;12(9):e0184435. doi: 10.1371/journal.pone.0184435 (PMC5590960; doi:10.1371/journal.pone.0184435)

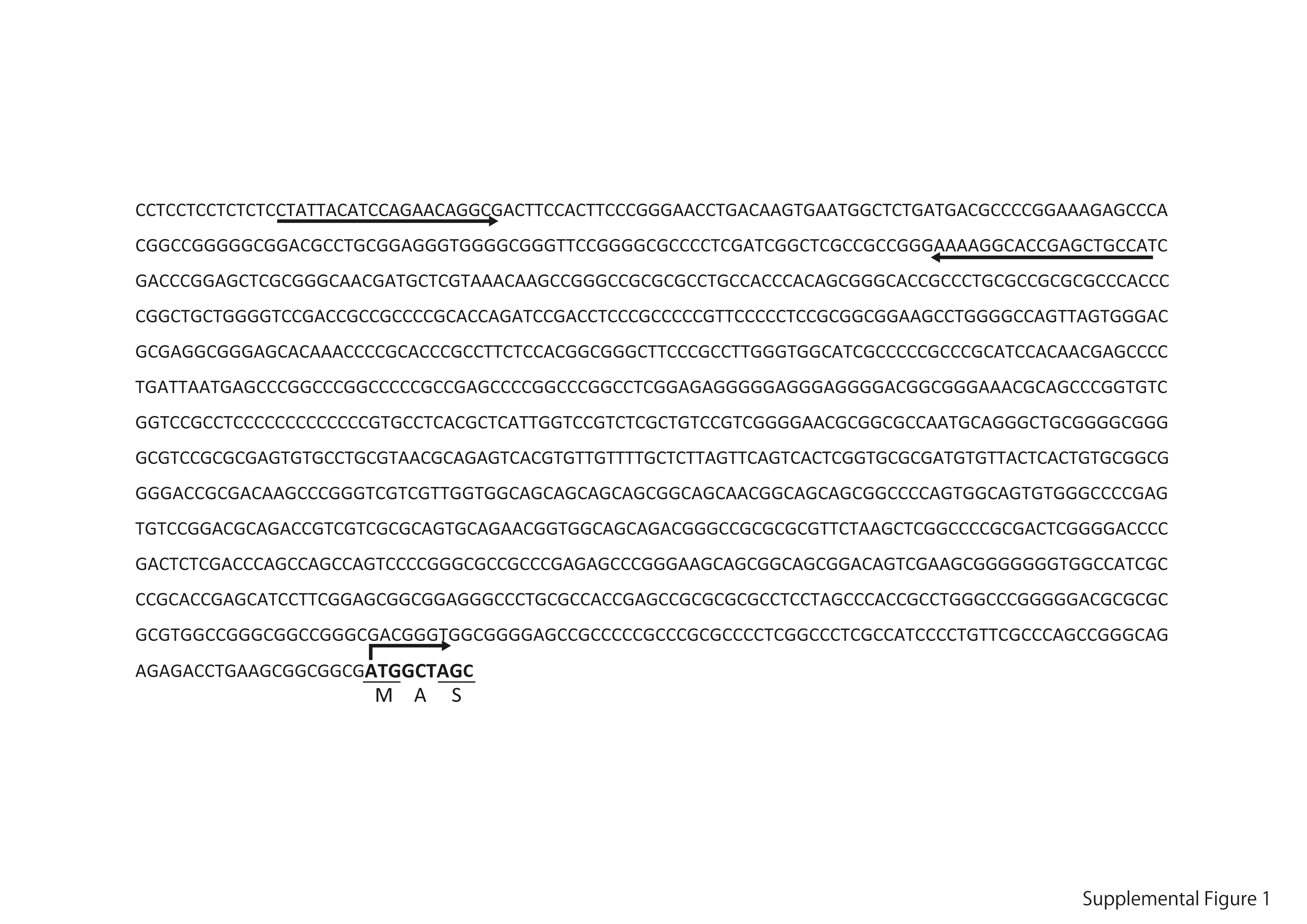

Supplement: S1 Fig — Arrows show the primers for ChIP analysis of H3K9/14 acetylation. (TIF) [file pone.0184435.s001.tif]

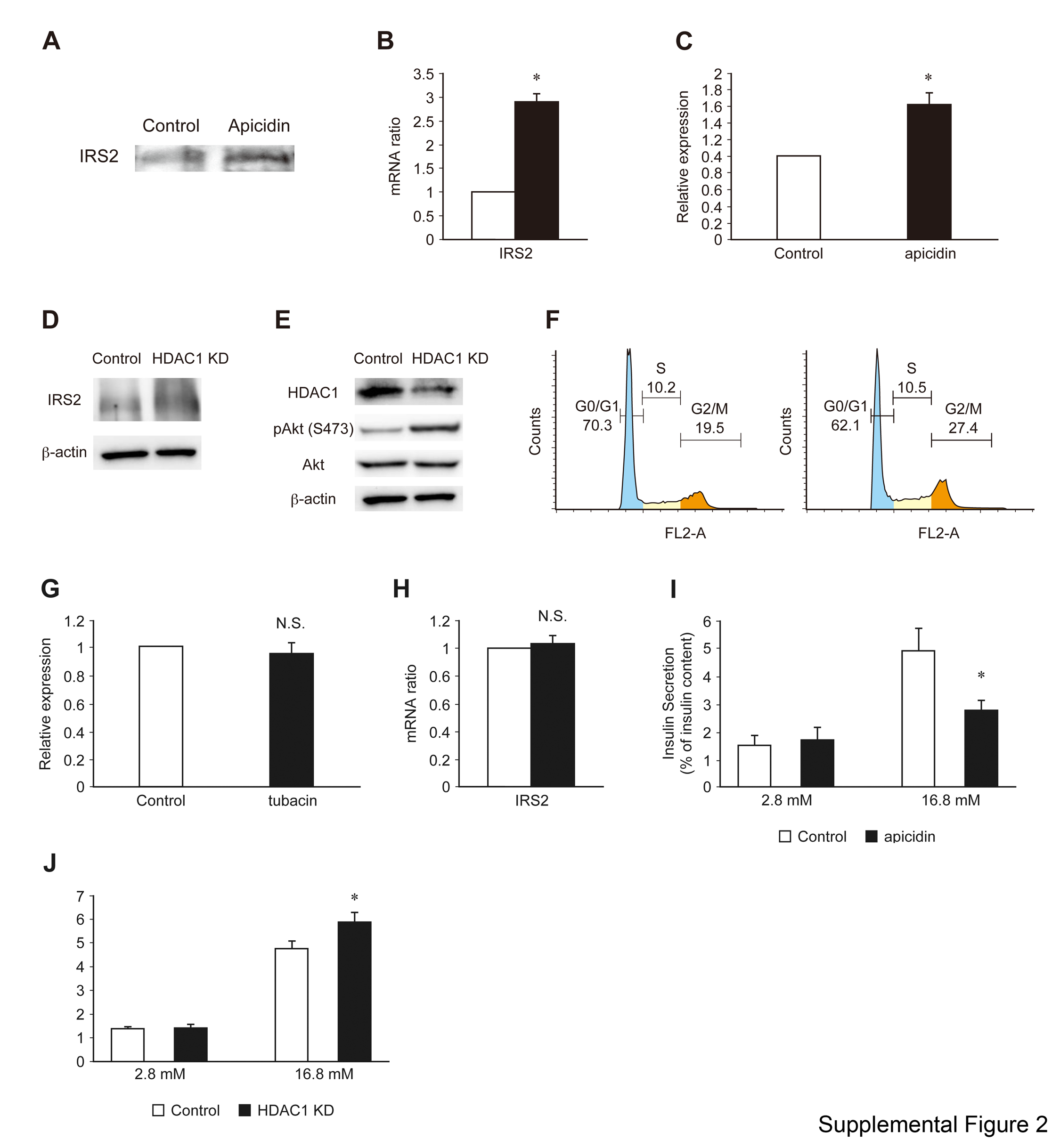

Supplement: S2 Fig — (A) Immunoblot analysis of IRS2 in MIN6 cells treated with the HDAC class I-specific inhibitor apicidin. (B) Quantitative real-time PCR analysis of Irs2 mRNA expression in control MIN6 cells (white bars) and those treated with apicidin (black bars). (C) ChIP-qPCR of H3K9/14 histone acetylation of the Irs2 promoter region in MIN6 cells treated with apicidin. (D, E) Immunoblot analysis of IRS2 (D) and insulin signaling proteins (E) in MIN6 cells treated with HDAC1 siRNA. (F) Representative cell cycle analysis in Lpn MIN6 cells with (right) or without (left) HDAC1 siRNA. (G, H) ChIP-qPCR of H3K9/14 histone acetylation of the Irs2 promoter region (G) and quantitative real-time PCR analysis of Irs2 mRNA expression (H) in MIN6 cells treated with the HDAC6-specific inhibitor tubacin. (I) Insulin secretion in response to the indicated concentrations of glucose from Lpn MIN6 cells with or without apicidin. (J) Insulin secretion in response to the indicated concentrations of glucose from Lpn MIN6 cells with or without HDAC1 siRNA. Data are represented as the mean ± SEM for 5 (A–H) and 6 (I, J) independent experiments. *P < 0.05. (TIF) [file pone.0184435.s002.tif]

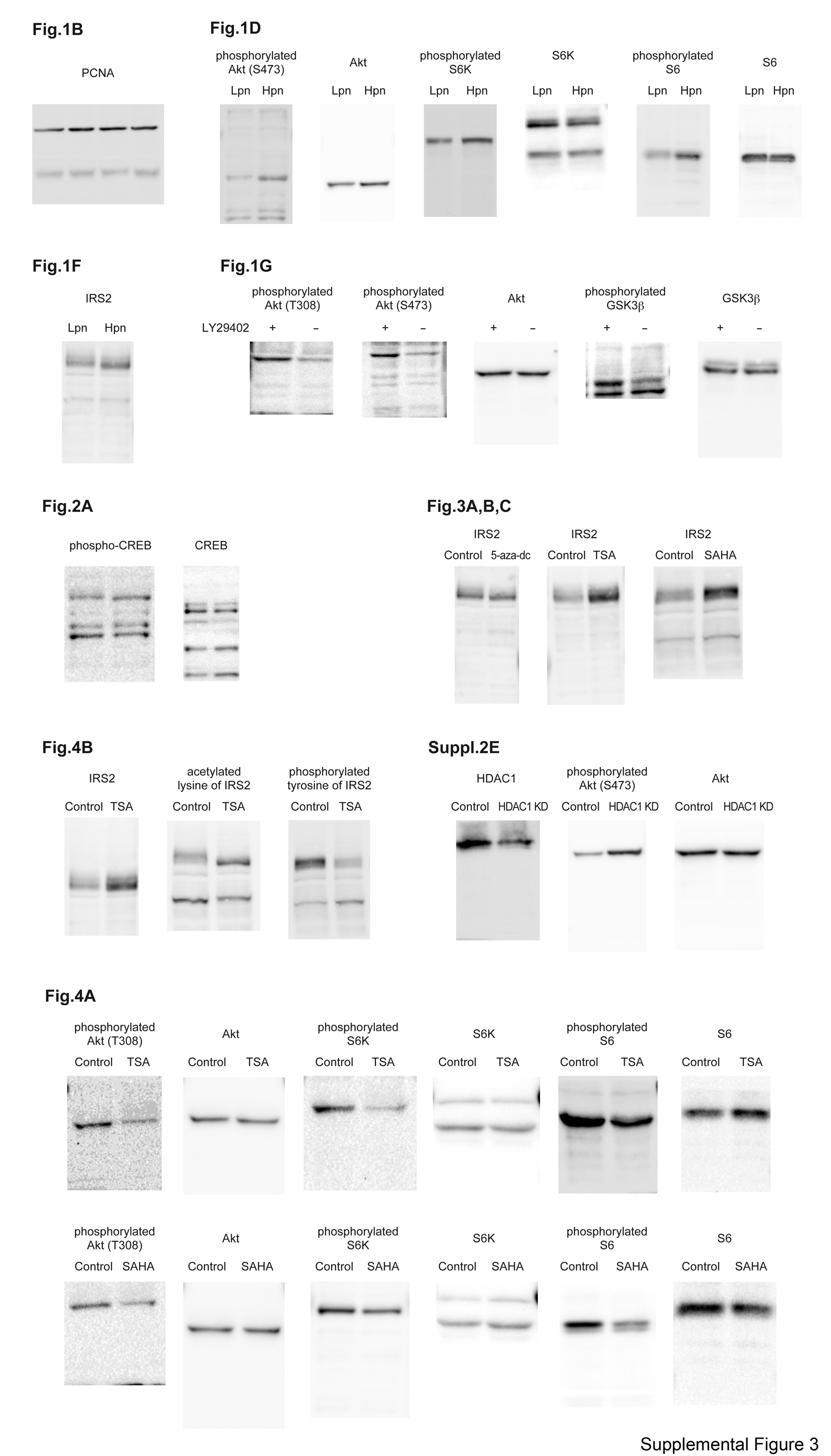

Supplement: S3 Fig — (TIF) [file pone.0184435.s003.tif]
